# Limited Efficacy of Nanoparticle-Assisted Electroporation for Membrane Permeabilization and Gene Electrotransfer

**DOI:** 10.3390/pharmaceutics17080964

**Published:** 2025-07-25

**Authors:** Tamara Polajžer, Matej Kranjc, Slavko Kralj, Maja Caf, Rok Romih, Samo Hudoklin, Federica Rocca, Damijan Miklavčič

**Affiliations:** 1 Faculty of Electrical Engineering, University of Ljubljana, Tržaška 25, SI-1000 Ljubljana, Slovenia; tamara.polajzer@fe.uni-lj.si (T.P.); matej.kranjc@fe.uni-lj.si (M.K.); 2 Department for Materials Synthesis, Jožef Stefan Institute, Jamova cesta 39, SI-1000 Ljubljana, Slovenia; slavko.kralj@ijs.si (S.K.); maja.caf@ijs.si (M.C.); 3 Faculty of Pharmacy, University of Ljubljana, Aškerčeva cesta 7, SI-1000 Ljubljana, Slovenia; 4 Institute of Cell Biology, Faculty of Medicine, University of Ljubljana, Vrazov trg 2, SI-1000 Ljubljana, Slovenia; rok.romih@mf.uni-lj.si (R.R.); samo.hudoklin@mf.uni-lj.si (S.H.); 5 Faculty of Bioengineering, University of Genova, Via All’Opera Pia 13, 16145 Genova, Italy; federica.elisa.rocca@gmail.com

**Keywords:** nanoparticles, electroporation, numerical model, permeabilization, gene electrotransfer

## Abstract

**Background/Objectives**: Nanoparticles (NPs) were previously explored as enhancers in electroporation due to their potential to locally amplify electric fields near cell membranes, with gold nanoparticles (AuNPs) in particular showing promise in improving membrane permeability and gene electrotransfer (GET). In this study, we systematically investigated the influence of NP properties—including size, shape, surface functionalization, and material—on electroporation efficacy. **Methods**: A combined approach using theoretical modeling and experimental validation was employed, encompassing numerical simulations, membrane permeabilization assays, transmission electron microscopy, and GET efficiency measurements. **Results**: Numerical results revealed that the presence of NPs alters local electric field distributions, but the amplification is highly localized, regardless of NP conductivity or geometry. Experimentally, only two out of six tested NP types produced a statistically significant, yet modest, increase in membrane permeability at one electric field intensity. Similarly, GET improvement was observed with only one NP type, with no dependence on concentration or functionalization. **Conclusions**: Overall, our findings demonstrate that NPs, under tested conditions, do not substantially enhance cell membrane permeability or GET efficacy. These conclusions are supported by both computational modeling and in vitro experiments.

## 1. Introduction

Pulsed electric field (PEF) treatment involves the application of short pulses of electric fields, also known as electroporation. Electroporation causes the formation of water pores, lipid peroxidation, and protein damage in the cell membrane [1]. These membrane changes increase the membrane’s permeability, allowing molecules that would otherwise be unable to pass through [2]. The changes (injuries) are transient and allow the cell to recover if not excessive. A transient increase in membrane permeability can be used for transfection, i.e., the introduction of foreign nucleic acids (RNA, DNA) into cells, resulting in genetically modified cells. The delivery of nucleic acids with PEF treatment is also known as gene electrotransfer (GET). GET is cost-effective, flexible, and eliminates the need for chemicals or viruses, making it a safe choice for clinical applications [3]. GET is investigated and used for monoclonal antibody production, DNA vaccination, and gene therapy, including CAR-T cell therapy and stem cell engineering [4,5,6,7]. In addition to permeabilizing the cell membrane, electroporation pulses provide an electrophoretic force that facilitates the transport of charged genetic material toward the membrane. This leads and enables contact between genetic material and the plasma membrane, which is believed to be a crucial step in successful GET [3,8,9,10,11,12]. Furthermore, the success of GET depends on achieving membrane permeability while maintaining high cell and tissue viability [3].

Nanoparticles (NPs) are defined as particles with a diameter of up to 100 nanometers and exhibit unique properties based on their size, chemical composition, and surface area [13]. They can be utilized for targeted drug delivery by loading drugs inside the NPs and attaching targeting ligands to their surface [14]. Membrane electroporation and permeability can be enhanced by adjusting pulse parameters such as pulse duration, pulse repetition rate, amplitude, and the number of pulses. In addition, both theoretical and experimental studies have suggested that the presence of NPs can further increase membrane permeability [15,16,17,18,19,20,21]. In recent years, NPs have demonstrated significant potential in biotechnology and medicine.

In electroporation-based treatments, the most commonly used NPs are gold nanoparticles (AuNPs). AuNPs can be synthesized in various shapes, including spherical, triangle, hexagon, and rod, and are known for their low toxicity [22,23]. In electroporation, AuNPs are typically used in their plain form (without drugs or ligands), as it is believed that their proximity to the cell membrane modifies the electric field. Lekner et al. theoretically demonstrated and proposed that the electric field to which cells are exposed, with the aim of electroporating them, can be amplified (by a factor of 100) using elongated micro-conductors such as prolate spheroids. These micro-conductors, e.g., AuNPs, significantly enhance the electric fields at their edges, enabling increased electroporation. Increased electroporation efficacy was initially demonstrated theoretically and later confirmed experimentally [15,16,17,18,19,20,21]. The first studies have shown that the presence of AuNPs can enhance membrane permeability when using impermeable dyes [15] and improve the outcome of gene electrotransfer (GET) [24]. However, the extent of improvement in permeability or GET efficacy depends on the size and concentration of the AuNPs. Larger AuNPs have been found to be more effective, suggesting that the properties of NPs influence electroporation efficacy [15,25].

To investigate the influence of NP properties on electroporation efficacy more systematically, we synthesized different NPs. The NPs used in our study varied in size, shape, functionalization, and material. In the study, we investigated the effect of various NPs on electroporation efficacy. This was first carried out using a theoretical approach with numerical modeling, followed by experimental research that involved membrane permeabilization, transmission electron microscopy, and assessing GET efficiency.

## 2. Materials and Methods

Chemicals needed for NPs: Bis(p-sulfonatophenyl)phenylphosphine (97%), citric acid monohydrate (99.5–100.5%), L-(+)-ascorbic acid (99.5–100.5%), O-(2-mercaptoethyl)-O′-methylpolyethylene glycol (2000), and silver nitrate (≥99.8%) were obtained from Sigma-Aldrich. A hydrogen tetrachloroaurate trihydrate (99.99%) and sodium hydroxide (98%) were acquired from Alfa Aesar. A hexadecyltrimethylammonium bromide was purchased from VWR, sodium borohydrate (98+%) from Acros Organics, sodium chloride (NaCl) (≥99.5%) from Fisher Scientific, and Tween 80 from Fisher Bioreagents. All chemicals were used as received without further purification.

Gold nanorod synthesis (AuNRs) and functionalization (AuNRs-PEG): The procedure for gold nanorod synthesis was based on the seed-mediated growth method described by Nikoobakht and El-Sayed [26]. Firstly, the fresh seed solution was prepared by mixing solutions of 5 mL (0.00050 M) hydrogen tetrachloroaurate (III) hydrate, 0.6 mL (0.010 M) sodium borohydride, and 5 mL (0.20 M) hexadecyltrimethylammonium bromide (CTAB). The sodium borohydride and hydrogen tetrachloroaurate (III) hydrate solutions were cooled before the synthesis began. Then, a CTAB solution was mixed with the hydrogen tetrachloroaurate (III) hydrate solution, and this mixture was vigorously stirred (1200 rpm) followed by quick addition of the sodium borohydride solution. Immediately after reductant addition, the solution color changed from yellow to brown. This seed solution was stirred for an additional 2 min and then left in the dark at 30 °C for 4 h. Meanwhile, solutions of 0.0040 M silver nitrate (3.83 mL), 0.0788 M ascorbic acid (1.786 mL), 0.001 M HAuCl_4_·3H_2_O (127 mL), and 0.2 M CTAB (127 mL) were prepared and placed in a water bath at 30 °C for one hour prior to AuNR synthesis. The mixture of AgNO_3_, CTAB, and HAuCl_4_·3H_2_O solutions was stirred at 1200 rpm. Upon rapid addition of the ascorbic acid solution, the solution’s color changed immediately from yellow to colorless. Subsequently, 0.306 mL of the seed solution was added, and the mixture was stirred for an additional 30 s before being left in a water bath at 30 °C in the dark overnight. Finally, the NPs were washed twice with distilled water using an ultracentrifuge at 20,000× *g* for 20 min to remove the excess CTAB. Then, the NPs were resuspended in distilled water. Subsequently, the synthesized AuNRs were pegylated following the synthesis protocol described by Liu et al. [27]. In brief, solutions of 2 vol% Tween 80, 0.1 M bis(p-sulfonatophenyl) phenylphosphine (BSSP, 25 µL), 1.6 mM O-(2-mercaptoethyl)-O′-methylpolyethylene glycol (MeO-PEG-SH, 63 µL), and 2 M sodium chloride (NaCl, 250 µL) were prepared and stirred for one minute. Then, the NP suspension was added to this mixture and left to shake overnight. After the reaction was complete, the pegylated gold nanorods (AuNRs-PEG) were washed with distilled water.

Spherical 10 nm gold NP synthesis (10-AuNPs) and their functionalization (10-AuNPs-PEG): The protocol for the synthesis of 10 nm-sized gold NPs was adapted according to Ojea-Jiménez et al. [28]. First, solutions of 0.066 M HAuCl_4_·3H_2_O (2 mL), 2.45 mM sodium hydroxide (102 mL), and 0.34 M sodium citrate (2.1 mL) were prepared. Then, the sodium hydroxide and sodium citrate solutions were transferred to a flask equipped with a condenser and heated in an oil bath at 112 °C until it reached boiling. Once the solution has boiled, the stirring speed was set to 800 rpm, and the HAuCl_4_·3H_2_O solution was rapidly added. Immediately after the addition, the solution turned black, and after a few minutes, it gradually changed to a wine-red color, indicating the formation of gold NPs. After the reaction was completed, the NP suspension was cooled to room temperature using an ice bath. The synthesized 10-AuNPs were then washed with distilled water. The synthesized 10-AuNPs were pegylated using the following protocol: the NP suspension was added dropwise to a vigorously stirred MeO-PEG-SH solution (0.125 mM, 20 mL) and left to react overnight. The next day, the synthesized 10-AuNPs-PEG were washed once with distilled water.

Spherical 50 nm gold NP synthesis (50-AuNPs) and their functionalization (50-AuNPs-PEG): The synthesis of larger, 50 nm spherical gold NPs was adapted from Bastús et al. [29]. First, aqueous solutions of sodium citrate (2.2 mM and 60 mM) and HAuCl_4_ (25 mM) were prepared. Gold seeds were then synthesized by heating 37.5 mL of 2.2 mM sodium citrate in a flask placed in an oil bath with a condenser until the solution reached boiling. Next, 0.25 mL of HAuCl_4_ was added while stirring vigorously at 800 rpm for one minute, leading to a color change to light pink. The flask containing the seed solution was left at room temperature to cool to 90 °C. Once cooled, the seeds were transferred back to the oil bath at the same temperature, and the mixing speed was adjusted to 600 rpm. A 0.25 mL aliquot of 25 mM HAuCl_4_ was added, with two additional additions at 10 min intervals (three additions in total). Following the final addition, a solution of 13.25 mL of distilled water and 0.5 mL of 60 mM sodium citrate was heated to 90 °C. Once at temperature, 13.75 mL of the previously synthesized NPs was introduced, followed by three consecutive additions of 0.25 mL HAuCl_4_ at 10 min intervals. This dilution and addition process was repeated four times in total. After the final synthesis step, the NPs were washed with distilled water to remove excess sodium citrate. The synthesized 50-AuNPs were pegylated using the same procedure as the smaller (10 nm) gold NPs. The pegylated 50-AuNPs were labeled as 50-AuNPs-PEG.

Silica NP synthesis: The synthesis method was performed following the Stöber method [30]. Briefly, 50 mL of water, 4.5 mL of NH_3_, and 50 mL of a previously prepared 0.27 M TEOS solution were mixed in a flask and stirred overnight. After synthesis was completed, the silica NPs were washed three times with distilled water. These NPs were labelled SiO_2_-NPs.

Characterization of NPs: Transmission electron microscopy (TEM) analyses were acquired using a Jeol 2100 TEM equipped with energy-dispersive X-ray spectroscopy (EDXS, JED 2300 EDS). For all synthesized NP types, TEM grids were prepared by depositing a few drops of the diluted sample onto the grid and allowing them to dry. NP size (from TEM images) distributions were analyzed using ImageJ software, version 1.53t, based on a large dataset (N = 500). Zeta potential measurements and hydrodynamic size determinations were performed using a Litesizer 500 (Zeta meter and DLS, Anton Paar, Graz, Austria). The NP suspensions were diluted to 250 µg/mL with HAM buffer and transferred to a cuvette for zeta potential measurements while DLS were measured in different solvents at 200 µg/mL.

**Figure 1:**
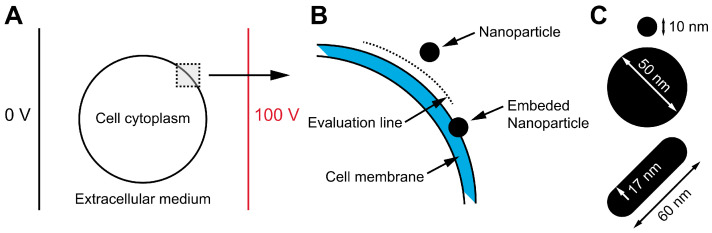
Schematic illustration of the numerical model designed to investigate the influence of NPs on the electric field distribution near the cell membrane. The model consists of a cell positioned between two electrodes, immersed in an extracellular medium (**A**). More specifically, the cell had a 5 nm-thick cell membrane, and an NP was placed 10 nm away from the membrane. The electric field was calculated along the evaluation line between the cell membrane and the NP. In addition, to account for potential interactions between the NPs and the cell membrane, the electric field amplification in the cell membrane was also assessed in a scenario where the NP was embedded in the membrane (**B**). Three different NPs (two spherical and one nanorod) were used for electric field assessment (**C**).

Numerical modeling: Finite element method (FEM) simulations were performed using the commercial software COMSOL Multiphysics, version 6.0, to analyze the impact of NPs on the electric field distribution when placed near the cell membrane (Figure 1). The two-dimensional numerical model represented a cell as a circle with a radius of 10 µm and a membrane thickness of 5 nm. Spherical NPs were modeled as circles with radii of either 10 nm or 50 nm, while the nanorod was represented as a capsule with a radius of 17 nm and a length of 60 nm. A constant voltage was applied between two parallel electrodes positioned at the left and right boundaries of the simulation domain. The NPs were placed 10 nm away from the cell membrane, i.e., the distance of the nearest point of NPs to the cell membrane was 10 nm. This spacing was the smallest distance at which numerical convergence of the model could be achieved. The amplification of the electric field was evaluated on the evaluation line (between the cell membrane and NPs) (Figure 1B) and inside the cell membrane. Furthermore, to account for potential interactions between the NPs and the cell membrane, the electric field amplification was also assessed in a scenario where the NP was embedded in the membrane, causing localized membrane thinning. The electrical properties used in the model were taken from and are listed in Table 1.

Cells: The Chinese hamster ovary cell line (CHO) was purchased from the European Collection of Authenticated Cell Cultures. Cells were grown in HAM F-12 growth medium (PAA, Oberosterreich, Austria) supplemented with 10% fetal bovine serum (FBS, Sigma-Aldrich, Saint Louis, MO, USA), L-glutamine (0.5% for CHO, 2% for H9c2) (StemCell, Vancouver, BC, Canada), penicillin/streptomycin (PAA, Oberosterreich, Austria), and 0.1% gentamycin (Sigma-Aldrich, Saint Louis, MO, USA) in an incubator at 37 °C with a controlled atmosphere (CHO at 5% CO_2_, H9c2 at 10% CO_2_). The human urinary bladder transitional carcinoma cell line (T24) was purchased from American Type Culture Collection (ATCC). Cells were grown in A-DMEM:F12 growth medium (Gibco, Thermo-Fisher, Waltham, MA, USA) mixed 1:1 and supplemented with 5% FBS (Gibco) and 4 mM Glutamax (Gibco). T24 cells were grown in incubator at 37 °C with a controlled atmosphere (5% CO_2_). For experiments, growth medium was removed and trypsin–EDTA (PAA, Oberosterreich, Austria) was added to detach the cells. After 3 min, fresh medium was added to inactivate the trypsin. The cell suspension was then centrifuged at 180× *g* for 5 min, the supernatant was removed, and the cells were resuspended in cell growth media with different concentrations of NPs (0, 50, 100, or 200 µg/mL) to 1 × 10^6^ cells/mL or, in the case of GET, 2 × 10^6^ cells/mL density. The cell suspension was incubated at 4 °C for 15 min, after which pulses were delivered.

Electric pulse applications: Pulsed electric field (PEF) was applied with prototype pulse generator L-POR V0.1 (mPOR, Ljubljana, Slovenia), described previously in Ref. [8]. Eight pulses of 100 µs duration with a 1 Hz repetition rate were delivered at different amplitudes. Pulse delivery was monitored by high-voltage differential probe HVD3605A (Teledyne LeCroy, Chestnut Ridge, NY, USA), current probe CP031 (Teledyne LeCroy, Rockland County, NY, USA), and high-definition oscilloscope HDO6000 (Teledyne LeCroy, Rockland County, NY, USA).

**Figure 2:**
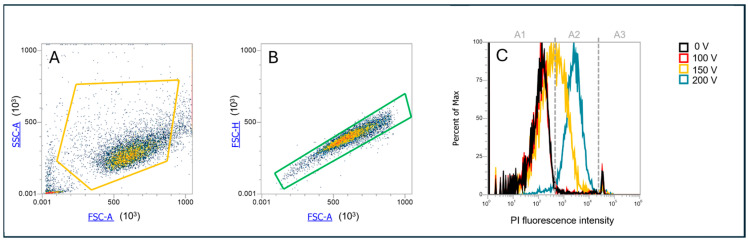
Gating strategy for determination of PI permeabilized cells. (**A**) Determination of cell population; (**B**) selection of only single cells, exclusion of doublets; (**C**) PI fluorescence histogram, with gates determining area A1—viable nonpermeabilized cells with a low PI signal, A2—viable permeabilized cells with a moderate PI signal, and A3—dead cells with a high PI signal.

Permeabilization assay: Prior to PEF application, cells were mixed with propidium iodide (Sigma-Aldrich, Saint Louis, MO, USA) to a final concentration of 100 µg/mL. A total of 150 µL of cell suspension was transferred to 2 mm aluminum cuvettes (VWR International, Radnor, PA, USA). Three minutes after the treatment, the cell suspension was removed from the cuvette, and the uptake of PI in the cells was analyzed by flow cytometer (Attune NxT; Life Technologies, Carlsbad, CA, USA) using a 488 nm blue laser and a 574/26 nm band-pass filter. The analysis of 10,000 events was performed by Attune Nxt software, version 5.3.0. On the dot plots of forward scatter and side scatter, the debris and clusters were excluded from the analysis (Figure 2A). Next, only single cells were selected for fluorescence analysis (Figure 2B) Fluorescence intensity histograms like in Ref. [31] were used to determine the percentage of live PI positive, i.e., permeabilized cells (Figure 2C). The sham control (0 V) was used to determine an area of live nonpermeabilized cells, i.e., with low PI fluorescence intensity (Figure 2C, A1), and an area of dead cells, i.e., with strong PI fluorescence intensity (Figure 2C, A3). Between them, there was an area for live permeabilized cells with moderate PI fluorescence intensity (Figure 2C, A2). Experiments for each data point were repeated three times.

Survival assay: After PEF treatment, cells were diluted in fresh growth medium and 2 × 10^4^ cells were transferred to wells in a 96-well plate (Techno Plastic Products AG, Trasadingen, Switzerland) and incubated at 37 °C for 24 h. According to the manufacturer’s instructions (CellTiter 96 AQueous One Solution Cell Proliferation Assay, Promega, Madison, WI, USA), 20 µL of MTS tetrazolium compound was added to the samples and incubated at 37 °C for additional 2 h, after which the absorbance of formazan (reduced MTS tetrazolium compound) was measured with a spectrofluorometer (Tecan Infinite M200, Tecan, Männedorf, Switzerland) at 490 nm. The percentage of viable cells was obtained by the normalization of sample absorbance to the absorbance of the control (0 V) with the same NP concentration.

Transmission electron microscopy (TEM): CHO and T24 cells in the absence and presence of NPs with (150 V—voltage resulting in membrane permeability changes) or without (0 V) electric pulse delivery, i.e., with or without electroporation treatment, were prepared for TEM analysis. Immediately after electroporation treatment, 150 µL of cell suspension was mixed with 150 µL fixative (2.5% glutaraldehyde in 0.1 M cacodylate buffer pH 7.2). After 5 min, three repetitions of each sample were merged into one sample to obtain a sufficient number of cells for analysis. The samples were centrifuged at 200× *g* for 5 min, the fixative was replaced by 1 mL of fresh fixative, and the samples were fixed for additional 1 h at room temperature. The samples were centrifuged (200× *g*, 5 min), the fixative was discharged, and the samples were washed in 0.33 M sucrose in 0.1 M cacodylate buffer overnight at 4 °C. Cells were post-fixed with the mixture of 1% OsO_4_ and 0.8% potassium ferrocyanide for 30 min at 4 °C in the dark. Samples were washed with H_2_O, en bloc stained with 2% uranyl acetate for 30 min at room temperature in the dark, washed, dehydrated in series of ethanol, and embedded in Epon (Sigma-Aldrich, USA). At 3 different locations from each sample, 60 nm-thick ultrathin sections were cut with ultramicrotome (EM UC6, Leica microsystems, Wetzlar, Germany), contrasted with lead citrate and uranyl acetate, and observed under a transmission electron microscope (CM100, Philips, Amsterdam, The Netherlands) running at 80 kV.

Gene electrotransfer (GET) assay: Gene electrotransfer was evaluated with 4.7 kb plasmid pEGFP-N1 (Clontech Laboratories Inc, Mountain View, CA, USA) encoding green fluorescent protein (GFP) under the control of a CMV promotor. Plasmid (pDNA) was amplified using Escherichia coli and isolated with the HiSpeed Plasmid Maxi Kit (Qiagen, Hilden, Germany). Plasmid concentration was spectrophotometrically determined at 260 nm. Prior to treatment, cells (with or without NPs) were mixed with the plasmid to obtain a concentration of 100 µg/mL of plasmid. A total of 150 µL of cell suspension with plasmid was transferred to a 2 mm cuvette, and immediately after the pulse application 40 µL of fetal bovine serum was added to the cells and incubated for another 5 min at 37 °C. Afterwards, the cells were diluted in fresh growth medium, and the sample was split for the two following assays—survival (as described above) and GET. For GET analysis, the cells were seeded in growth medium in a 24-well plate (Techno Plastic Products AG, Trasadingen, Switzerland) for 24 h at 37 °C, 5% CO_2_. Afterwards, the cells were harvested and resuspended in 150 µL of phosphate buffer saline (1x PBS), and the percentage of GFP-positive cells was detected using flow cytometer Attune NxT (ThermoFisher Scientific, Waltham, MA, USA) with a blue laser at 488 nm and a 530/30 nm bandpass filter. For every sample, 10,000 events were recorded. Data obtained were analyzed using Attune NxT software. Overall gene electrotransfer was calculated multiplying the % of GFP positive cells and survival after GET. To determine the experimental voltage point for GET, six voltages (0, 175, 200, 225, 250, 275 V) were tested for GET in the absence of NPs. The voltage resulting in the best overall GET (250 V) was used in GET experiments with NPs.

Statistical analysis: All experiments except for TEM were repeated at least three times. The results are shown as mean ± SD. Statistical analysis was performed using SigmaPlot 11.0 (Systat Software, San Jose, CA, USA). Statistically significant differences (* *p* < 0.05) were determined by one-way ANOVA test and the Holm–Sidak post-hoc test.

## 3. Results

### 3.1. Characterization of Nanoparticles

**Figure 3:**
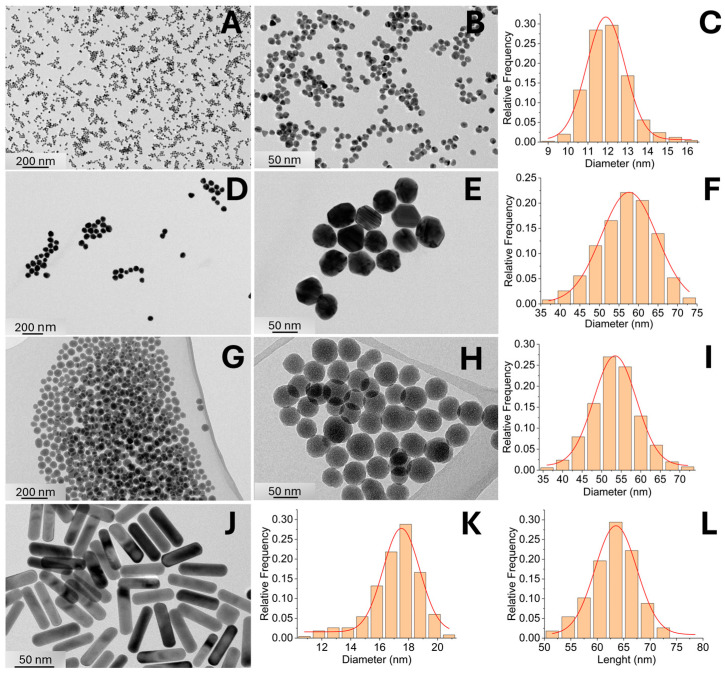
Characterization of synthesized NPs. 10-AuNP (**A**–**C**), 50-AuNPs (**D**–**F**), SiO_2_-NPs (**G**–**I**), and Au-NRs (**J**–**L**), including representative TEM images and size distributions graphs based on TEM image analysis.

The synthesized NPs were characterized using transmission electron microscopy (TEM), and their size distributions were analyzed by measuring particle dimensions from TEM images using ImageJ software (Figure 3 and Table 2). Modifying the synthesis protocol enabled the production of NPs with relatively narrow size distributions. The mean sizes of 10-AuNPs and 50-AuNPs were (12 ± 1) nm and (57 ± 7) nm, respectively. Gold nanorods with a mean diameter of (17 ± 2) nm and a length of (63 ± 4) nm, achieving an average aspect ratio of 3.7, were synthesized to investigate the influence of NP shape (Table 2). For experiments with conductive NPs, we used gold NPs either stabilized with citrate, exposing carboxyl groups, or functionalized with PEG, exposing methoxy groups. Citric acid is a small organic molecule that prevents NP agglomeration in complex media through electrostatic repulsion. Due to its small size, it allows NPs to come into close proximity to the cell membrane (hydrodynamic sizes are listed in Table 3). In contrast, PEG-functionalized NPs provide steric stabilization, as the long PEG chains (2 kDa) sterically repel NPs from each other and from the cell membrane. Citrate-stabilized gold NPs (10-AuNPs) have a zeta potential of (−11.6 ± 0.3) mV, whereas PEG-functionalized NPs (10-AuNPs-PEG) have a zeta potential of (−5.5 ± 1.5) mV. To investigate the effect of NP size, we also synthesized larger spherical gold NPs stabilized with either citrate (50-AuNPs) or PEG (50-AuNPs-PEG). The zeta potential values of these large NPs were similar to those of the smaller ones, with 50-AuNPs and 50-AuNPs-PEG exhibiting zeta potentials (−9.7 ± 0.5) mV and (−3.3 ± 0.7) mV, respectively.

Gold nanorods (Au-NRs), unlike other tested NPs, are synthesized in the presence of a relatively high concentration of hexadecyltrimethylammonium bromide (CTAB), which is toxic to cells and therefore should be eliminated and then replaced with a biocompatible alternative. In our study, we used PEG for this purpose, applying the same PEG molecule for functionalization as for spherical gold NPs. PEG functionalization replaces CTAB on the nanorod surface, making it suitable for experiments on living cells. Zeta potential measurements and determination of hydrodynamic sizes were not performed for Au-NRs or PEG-functionalized gold nanorods (Au-NRs-PEG), as the technique assumes a spherical particle shape, rendering the results unreliable for anisotropic structures. As for a control NP experiment to evaluate the effects of different NP conductivities in the electroporation experiments on the cells, we also synthesized non-conductive SiO_2_-NPs with a mean size distribution of (54 ± 6) nm and a zeta potential value of −10.9 ± 0.6 mV. However, all types of spherical gold NPs exhibited good colloidal stability in all tested media, as their mean hydrodynamic sizes consistently remained below 100 nm, with no peaks at larger sizes—indicating the absence of uncontrolled aggregation (Table 3).

### 3.2. Numerical Modeling

**Figure 4:**
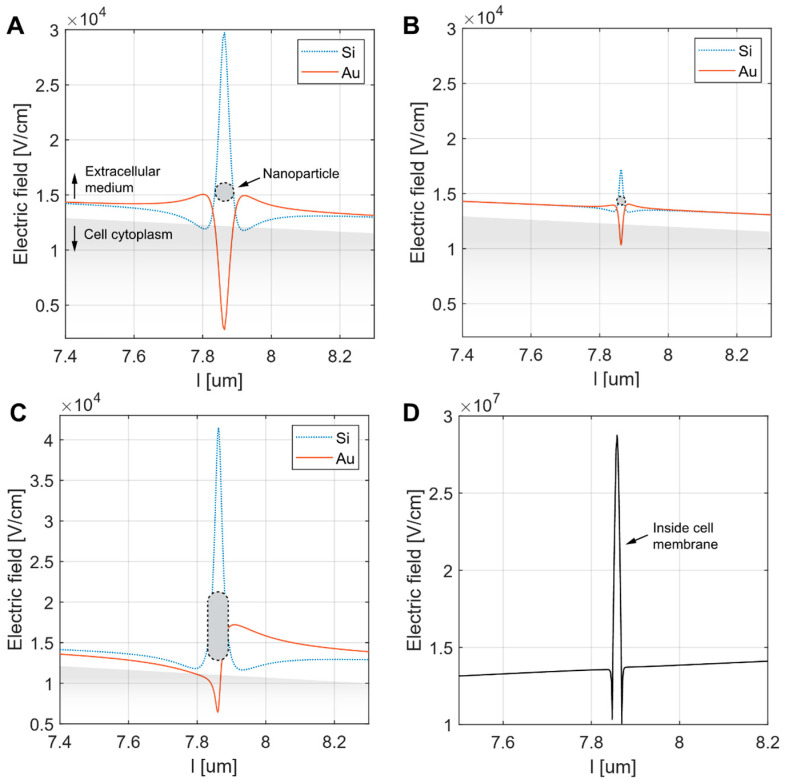
Simulation of the electric field along the evaluation line positioned between the cell membrane and the NP. The NPs considered were either spherical ((**A**)—radius 50 nm, (**B**)—radius 10 nm) or elongated ((**C**)—major axis 50 nm, minor axis 10 nm). The electric field was calculated for both silica (Si_2_O) and gold (Au) NPs of all types. This included the Si nanorod configuration, for which only the Au version was available in the experimental part of the study. In each panel, the NP position (indicated by a black dotted line) and the cell cytoplasm (shaded grey) are shown for illustrative purposes. Panel (**D**) shows the increase in the membrane electric field resulting from the embedded 50 nm gold NP inside the membrane. Note the different range of electric field in D.

The numerical calculation results reveal distinct electric field distributions influenced by the presence of NPs and their shape, size, and material properties, i.e., whether conductive or nonconductive. For spherical NPs, the electric field magnitude increased directly beneath the center of the NPs with NP size, as evidenced by the more pronounced peak in Figure 4A (50 nm radius) compared to Figure 4B (10 nm radius). In both cases, Si NPs generated higher electric field intensities than AuNPs directly beneath the center of the NPs. This is due to a higher current density “concentrating” in the narrow gap between the Si NP and the low-conductivity cell membrane, which increases the electric field intensity in accordance with Ohm’s law. The elongated NP (Figure 4C) exhibited the most significant electric field enhancement, particularly for Si, where a sharp peak was observed beneath the NP center. Nevertheless, the electric field dropped rapidly when moving away from the NP. In the case of the elongated AuNP, an asymmetric electric field distribution was observed, attributed to the directional flow of electric current: entering through the NP tip facing the cell membrane and exiting through the opposite tip. In all configurations, the increase in electric field inside the cell membrane, however, was found to be negligible, with a maximum increase of less than 0.3%. A significant increase in the electric field within the membrane was only observed in the case of an embedded NP due to membrane thinning. For example, a 50 nm NP embedded 2 nm into the membrane resulted in a more than double (210%) increase in the membrane electric field (Figure 4D).

### 3.3. Electron Microscopy

**Figure 5:**
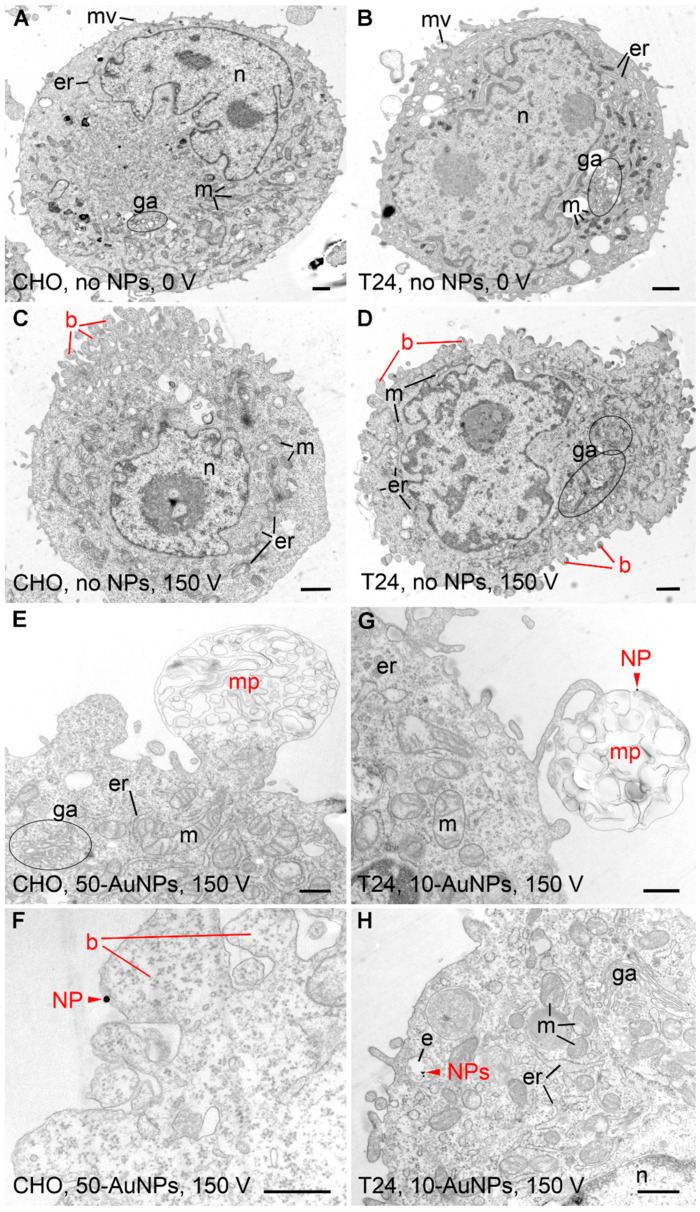
Ultrastructure of CHO and T24 cells after electroporation. (**A**,**B**) Control CHO and T24 cells exposed to no NPs (NPs). Ultrastructure of both cell types was normal, with visible nucleus (n), mitochondria (m), endoplasmic reticulum (er), Golgi apparatus (ga), and microvilli (mv). (**C**,**D**) CHO and T24 cells exposed to 150 V pulse, yet no NPs. Blebbing of the plasma membrane (b) was seen in both cell types. (**E**,**F**) CHO cells exposed to 150 V pulse in the presence of 50 nm gold NPs. (**G**,**H**) T24 cells exposed to 150 V pulse in the presence of 10 nm gold NPs. Membrane protrusions (mp) and occasionally NPs (red arrowhead—NPs) were seen in both cell types. NPs (red arrowhead—NPs) were located on the luminal side of the blebbing plasma membrane and within the endosomes (e) inside the cell. Scale bar for (**A**–**D**) is 1 µm and for (**E**–**H**) is 500 nm.

We analyzed the ultrastructure of cells and estimated the proximity/binding of NPs to the plasma membrane (Table 4). In CHO and T24 cells without NPs and without electroporation treatment (0 V), all cell compartments (nucleus, endoplasmic reticulum, Golgi apparatus, mitochondria) were well preserved. Both cell types had some microvilli on their surface (Figure 5A,B). No changes or damage to cell compartment morphology could be seen in any group with NPs. Some blebbing at the plasma membrane was observed regardless of NP type; however, it was more pronounced in groups with electroporation (150 V) treatment (Figure 5C,D). If NPs were found to be positioned less than 5 nm from the plasma membrane, they were considered to be attached to it. According to this criterion, NPs were attached to the plasma membrane in all groups of gold NPs except in CHO group 50 nm 50-AuNPs (0 V), T24 groups 10-AuNPs-PEG (0 V) and AuNRs-PEG (0 V), CHO and T24 group 10-AuNPs-PEG (150 V), and CHO and T24 group AuNRs-PEG (150 V). AuNRs-PEG were detected only on the plasma membrane of a single CHO cell without electroporation. SiO_2_-NPs were detected close to cells but never attached to the plasma membrane, i.e., being closer than 5 nm. Since permeabilization assays showed statistically significant differences in CHO cells treated with 150 V between the 50-AuNPs and no-NP groups, we focused on these two groups. On CHO cells with 50-AuNPs treated with 150 V, we detected large membrane protrusions containing membrane debris (Figure 5E) and blebs with NPs (Figure 5F) attached to the plasma membrane. Such structures were also observed on T24 cells with 10-AuNPs and electroporation (Figure 5G,H). This later group was also characterized by more NPs attached to their plasma membrane compared to other groups. In addition, we observed a small number of 10-AuNPs in the endosomes of these cells (as well as without electroporation treatment in T24 and CHO), pointing to some limited endocytosis (Figure 5H).

### 3.4. In Vitro Assessment of Nanoparticle Effect on Cell Membrane Permeabilization and Survival

To test NP cell cytotoxicity and determine nontoxic NP concentration for experiments, three concentrations—0, 100 and 200 µg/mL—of 50-AuNPs were tested. CHO cells were mixed with NPs and incubated for 24 h at 37 °C and 5% CO_2_. Afterwards, survival was assessed with an MTS assay. Results show that NPs up to 200 µg/mL were not toxic, as the viability was similar to the control—0 µg/mL, e.g., where NPs were not added (100% for 0 µg/mL and 104 ± 8.4% for 200 µg/mL). For NPs to have the most significant effect on electroporation efficacy, the highest concentration of 200 µL/mL of NPs was chosen for further experiments.

**Figure 6:**
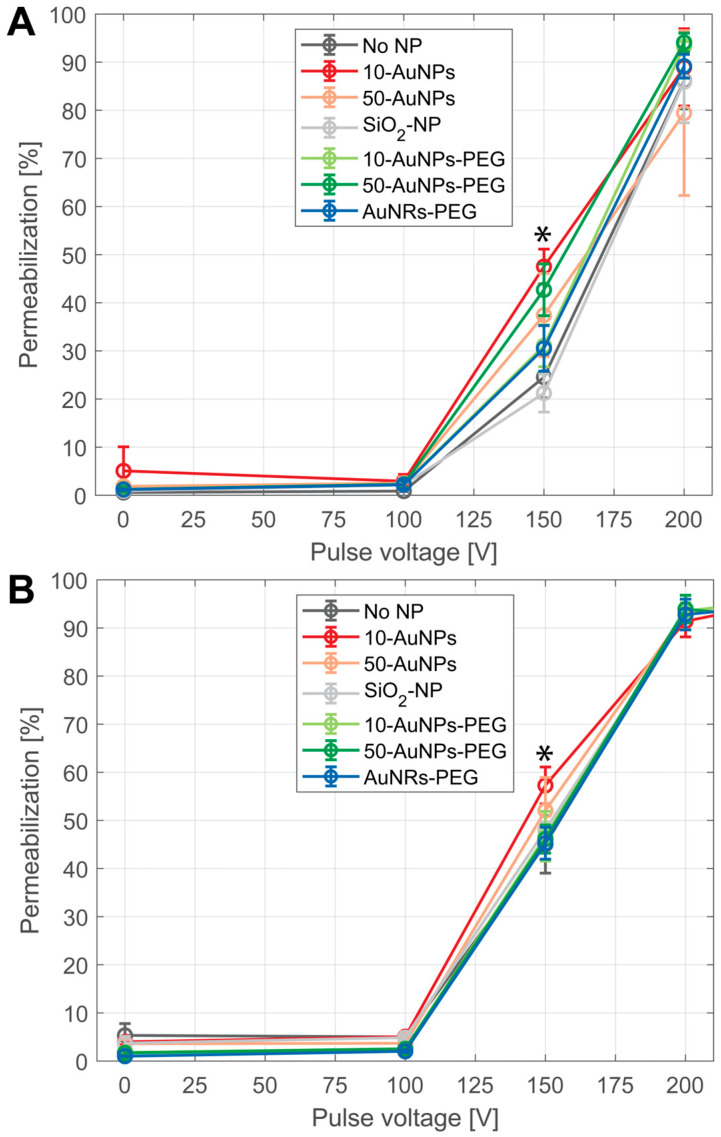
Membrane permeabilization after PEF treatment on (**A**) CHO and (**B**) T24 cells in the presence of NPs. Different colors represent use of different NPs: black—without NPs (no NP), red—10-AuNPs, orange—50-AuNPs, grey—SiO_2_-NPs, light green—10-AuNPs-PEG, dark green—50-AuNPs-PEG, and blue—AuNRs-PEG. Asterisks (*) mark a point of statistically significant increase between no NPs and 10-AuNPs in CHO, between no NPs and 50-AuNPs in the CHO cell line, and between no NPs and 10-AuNPs in the T24 cell line.

PI was used as a marker of cell membrane permeabilization due to electroporation. The results show that the presence of NPs did not change the threshold for electroporation; nor was the amplitude needed to achieve >90% of permeabilization (Figure 6). Differences in permeabilization efficiency were observed only in the middle part of the permeability curve, at 150 V. In the CHO cell line (Figure 6A), only 10-AuNPs and 50-AuNPs resulted in a statistically significant increase in permeability (*p* < 0.05) compared to treatment without NPs. However, there was no statistical significance due to the size of the NPs, i.e., between 10 and 50 nm (10-AuNPs and 50-AuNPs). Furthermore, we observed no increase in permeability efficacy compared to the treatment without NPs when PEG-functionalized AuNPs were used (10-AuNPs-PEG and 50-AuNPs-PEG). No difference in efficacy was observed when different shapes (spherical and nanorod) of NPs were compared (50-AuNPs-PEG and AuNRs-PEG). In the T24 cell line (Figure 6B), the differences between the presence and absence of NPs in the PEF treatment were even less evident, as only spherical 10-AuNPs resulted in increased permeabilization efficacy at 150 V compared to treatment without NPs. NP properties like size, shape, or functionalization or conductivity did not have any effect on increased membrane permeabilization. In both cell lines, SiO_2_-NPs, which were the only nonconductive NPs used, resulted in a permeabilization efficiency similar to that of the treatment without NPs.

### 3.5. Gene Electrotransfer (GET) with Nanoparticles

**Figure 7:**
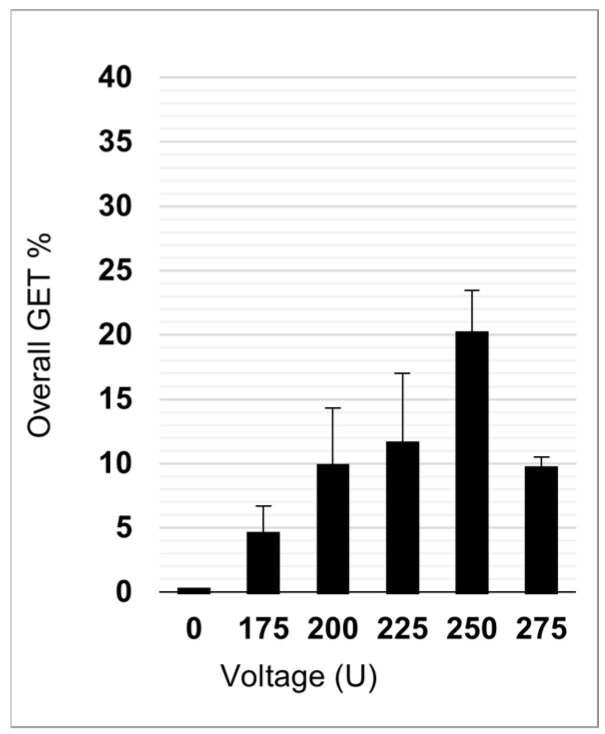
Overall GET efficacy without NPs at different electroporation voltages.

Preliminary GET experiments were performed in the absence of NPs (0 µg/mL) with 100 µg/mL of pEGFP-N1 plasmid (Figure 7). The most successful GET parameter was found with 250 V, and it was therefore chosen for GET experiments with NPs.

**Figure 8:**
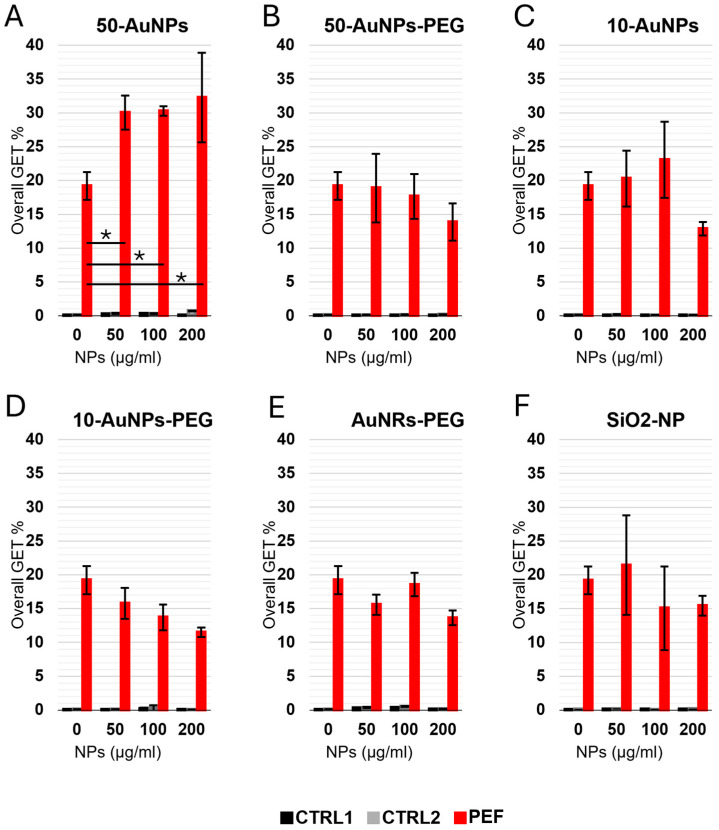
Overall GET in CHO cells performed with different NP concentrations. (**A**) 50-AuNPs, (**B**) 50-AuNPs-PEG, (**C**) 10-AuNPs, (**D**) 10-AuNPs-PEG, (**E**) AuNRs-PEG, (**F**) SiO_2_-NPs. Asterisks (*) mark a statistically significant increase. Each treatment had two controls—one with (black) and one without (gray) plasmid, both in the absence of the treatment (0 V), while the PEF treatment is shown in red.

GET with NPs and 100 µg/mL of pEGFP-N1 plasmid was performed with four different NP concentrations: 0 (without NPs), 50, 100, and 200 µg/mL (Figure 8). Each NP concentration had two controls: CTRL1 and CTR2. Both CTRLs were without PEF treatment (0 V); however, the plasmid was absent in CTRL1 and present in CTRL2. This showed that the presence of plasmid and/or NPs in the absence of electric pulses did not lead to a successful GET (Figure 8). Looking at GET when electric pulse (250 V) was applied, the only statistically significant (*p* < 0.05) increased GEF efficacy compared to GET without the presence of NPs was when 50-AuNPs were used. Interestingly, GET efficacy was independent of NP concentration, as there was no statistically significant difference in GET between 50, 100, and 200 µg/mL of NPs. Interestingly, other NPs (size, shape, functionalization, or conductivity) did not have an effect on GET. Furthermore, we could even observe a trend of reduced GET efficacy with an increase in NP concentration (although not statistically significant). The lower GET efficacy reflects a lower number of transfected cells, i.e., GFP-positive cells, as survival was not affected due to NPs or PEF (Supplementary Figures S1 and S2).

## 4. Discussion

It has previously been suggested theoretically and demonstrated experimentally that NPs can facilitate electroporation: GET, drug delivery/electrochemotherapy, and even the extent of the area of tissue being ablated by irreversible electroporation has been reported [18,20,24,25,32]. The NPs that have mainly been used in these experiments were carbon nanotubes and conductive/metal NPs like gold and platinum NPs. The underlying hypothesis in the reported studies was that conductive NPs in the electric field locally amplify the electric field, and thus, if NPs are close to the cell plasma membrane, they facilitate electroporation and achieve meaningful results at lower electric fields. Theoretical predictions are, however, based on continuum models, meaning that NP functionalization and interaction with plasma membrane are (over)simplified and idealized. Even though it has been calculated that the local electric field in the immediate vicinity of conductive NPs is increased by a factor of 3 [16,33], this amplification drops rapidly with the distance from the NP. It is also not clear how functionalization and coating/surface properties of NPs affect the interaction of NPs with the plasma membrane and influence electric field amplification. In the present study, we systematically explored the interaction of different NPs and cell membranes using theoretical and experimental approaches and assessed membrane permeabilization and the introduction of large molecules like plasmid DNA (pDNA) encoding for green fluorescent protein (GFP) by PEF, causing membrane electroporation.

The theoretical basis for the observed enhanced electroporation in previously reported studies was ascribed to local amplification of the electric field caused by conductive NPs [16,17,19]. However, to exert this amplification in the cell membrane, NPs must be close to the plasma membrane—how “close” to the membrane NPs can be becomes inapproachable by continuum models. Additionally, different coarse-grain molecular dynamics models compared to all atomistic models give different results with respect to NP interaction with the membrane due to different force fields used [34]. Our model showed that the enhancement of the electric field depended on the material, i.e., the electric properties of NPs (conductive vs. non-conductive), their geometry (spheres and rods), and their proximity to the membrane. However, the amplification of the electric field by NPs was small and very limited in distance for both conductive and nonconductive NPs. Even for the configuration that amplified the electric field most (up to 3 × 10^4^ V/cm in case of 10 nm spherical NP), the amplification was negligible compared to the transmembrane voltage (TMV) of 100–500 mV across the membrane, which resulted in an electric field strength of 10^6^ V/cm. NP electric field amplification thus represents only a fraction of the electric field presented across the membrane, which represents less than 0.3%.

Furthermore, additional calculations indicated that significant amplification of the electric field within the membrane could occur only when the NP was partially embedded in the membrane, leading to local membrane thinning, which could potentially explain the results of other studies, where the effect on NPs was more pronounced. For example, calculations show that a 50 nm gold (Au) NP embedded 2 nm into the membrane resulted in a 210% increase in the membrane’s electric field (Figure 4D). Interestingly, such thinning of the membrane was also suggested by molecular dynamics simulations [34]. To assess membrane thickness and the distance of differently functionalized NPs from the plasma membrane, we used electron microscopy. The electron microscope did not allow us to distinguish between NPs that were up to 5 nm away from membrane and NPs embedded in the plasma membrane. The same was true for membrane thinning. Therefore, the true distance from the membrane and membrane thinning remains undetermined. Additionally, our in vitro results did not show a clear effect of NPs on membrane permeabilization or GET, which further suggests that membrane thinning likely did not occur in our study.

To investigate the amplification of the electric field by NPs, first membrane permeability thus membrane permeability for PI was investigated. Only two (spherical non-PEG-functionalized) out of all six types of NPs tested resulted in increased membrane permeabilization. This small yet statistically significant increase was present in only one experimental electric field point, i.e., at 150 V, while in our previous study this was observed across the whole range of electric field, resulting in a shift in the permeability curve [15]. In a past study, we used commercially available NPs, while in the present study, we synthesized our own NPs of different types and functionalized them with PEG, achieving more general results. The difference in behavior between PEG-functionalized and non-functionalized NPs in this study could be due to protein corona formation. PEG-functionalized NPs are known to resist nonspecific protein adsorption and protein corona formation due to their hydrophilic and “stealth” surface properties [35,36]. The PEG chains introduce steric hindrance, creating a physical barrier that prevents close interaction between the NPs and the cell membrane. This steric repulsion likely limits the ability of PEG-functionalized NPs to approach or adsorb onto the membrane surface, thereby reducing their potential to locally enhance the electric field during electroporation. In contrast, citrate-stabilized AuNPs, which carry a more negative zeta potential (−11.6 mV for 10-AuNPs compared to −5.5 mV for 10-AuNPs-PEG), showed greater enhancement of membrane permeabilization. While we did not directly quantify NP–membrane association, the difference in zeta potential may indicate a stronger electrostatic interaction between citrate-coated NPs and the cell membrane, potentially allowing for closer proximity and more effective electric field enhancement. Additionally, the presence or absence of a protein corona could influence NP–cell interactions [37,38]. PEG-functionalized NPs, due to their surface chemistry, are less prone to forming a protein corona—even in protein-containing media—whereas citrate-coated NPs may adsorb proteins from the surrounding environment [39]. This protein corona could mediate interactions with the cell membrane, potentially facilitating closer contact and enhancing permeabilization [35,36]. Based on the obtained experimental results, NPs did not increase permeabilization efficacy, which is also in line with our numerical calculations.

Furthermore, the introduction of large biomolecules such as plasmid DNA (pDNA) encoding green fluorescent protein (GFP) was assessed. Both the cell membrane and the plasmid DNA carry negative charges, resulting in natural electrostatic repulsion and limited interaction in the absence of an electric field. NPs possess a negative zeta potential; however, functionalization with polyethylene glycol (PEG) reduces the magnitude of this negative charge, zeta potential, which could potentially improve gene electrotransfer (GET) efficacy. Contrary to expectations, the experimental results did not show a significant difference in GET efficiency based solely on zeta potential differences among the various NP types. The measured difference in zeta potential between non-functionalized and PEG-functionalized NPs was relatively small. All zeta potential measurements were performed in the same buffer, which contained salts, ions, and organic molecules that could have shielded the absolute values observed, compared to measurements conducted in pure water. Interestingly, GET outcomes mirrored cell permeability trends: Only one type of spherical, non-PEG-functionalized NP demonstrated improved GET efficiency. Furthermore, GET efficacy appeared independent of NP concentration, suggesting that, overall, NPs—regardless of conductivity or surface functionalization—do not significantly enhance GET efficiency.

## 5. Conclusions

In this study, we show that the combination of NPs and pulsed electric field does not contribute to increased efficacy of electroporation. This conclusion was reached with both numerical calculations and in vitro cell experiments for introducing small reporter molecules and large nucleic acid. It seems that NPs do not function as nanoelectrodes in electroporation, as they lack the capacity to increase electric fields sufficiently to significantly increase membrane permeabilization and improve the introduction of large molecules.

## Figures and Tables

**Table 1:** Electrical properties of the cell cytoplasm, cell membrane, extracellular media, and Au/SI NPs (values taken from Ref. [25]).

	Electrical Conductivity (S/m)	Relative Permittivity (-)
Cell cytoplasm	0.2	1
Cell membrane	5 × 10^−7^	11.7
Extracellular media	1.5	1
AuNP	4.11 × 10^7^	1
SiO_2_ NP	1 × 10^−3^	1

**Table 2:** Characteristics of the synthesized NPs used in our study.

Nanoparticles	Material	Core Size (nm) *	Shape	Surface	Zeta Potential (mV)
50-AuNPs	Gold	57 ± 7	Spherical	COOH	−9.7 ± 0.5 mV
10-AuNPs	Gold	12 ± 1	Spherical	COOH	−11.6 ± 0.3 mV
50-AuNPs-PEG	Gold	57 ± 7	Spherical	PEG	−3.3 ± 0.7 mV
10-AuNPs-PEG	Gold	12 ± 1	Spherical	PEG	−5.5 ± 1.5 mV
AuNRs-PEG	Gold	63 ± 4 length 17 ± 2 diam	Elongated	PEG	/
SiO_2_-NPs	Silica	54 ± 6	Spherical	-OH	−10.9 ± 0.6 mV

* Size is determined by TEM image analysis.

**Table 3:** Hydrodynamic sizes of spherical AuNPs (200 µg/mL) in distilled water, HAM buffer, and DMEM medium, as determined by dynamic light scattering (DLS) using intensity distribution.

Nanoparticles	Solvent	Hydrodynamic Size (nm)
10-AuNPs	Distilled water	43.8 ± 0.4
10-AuNPs-PEG	Distilled water	52.3 ± 1.1
50-AuNPs	Distilled water	64.1 ± 1.0
50-AuNPs-PEG	Distilled water	75.9 ± 1.9
10-AuNPs	DMEM	75.7 ± 3.7
10-AuNPs-PEG	DMEM	75.5 ± 2.6
50-AuNPs	DMEM	90.4 ± 3.2
50-AuNPs-PEG	DMEM	76.4 ± 0.1
10-AuNPs	HAM	78.3 ± 0.8
10-AuNPs-PEG	HAM	85.4 ± 3.7
50-AuNPs	HAM	88.9 ± 3.0
50-AuNPs-PEG	HAM	76.5 ± 2.0

**Table 4:** List of observations under TEM. * = endocytosis, ND = no sample, - = no NPs closer than 5 nm from the plasma membrane, + = NPs attached to the plasma membrane, X = large membrane protrusions with membrane debris.

	CHO		T24	
	0 V	150 V	0 V	150 V
No NP	-	-	-	-
10-AuNPs	+ *	+	+ *	+ *, X
50-AuNPs	+	+, X	+	+
SiO_2_-NPs	-	-	-	-
10-AuNPs-PEG	ND	-, X	-	-
50-AuNPs-PEG	-	+	+	+
AuNRs-PEG	+, X	-	-	-

## Data Availability

Data are available from the corresponding author on request.

## Supplementary Materials

The following supporting information can be downloaded at: https://www.mdpi.com/article/10.3390/pharmaceutics17080964/s1, Figure S1: GFP-positive cells in GET treatment on CHO cells performed with different NP concentrations.; Figure S2: Survival in GET treatment on CHO cells performed with different NP concentrations.
