# Supplementary material for: Limited Efficacy of Nanoparticle-Assisted Electroporation for Membrane Permeabilization and Gene Electrotransfer
Source: Pharmaceutics. 2025 Jul 25;17(8):964. doi: 10.3390/pharmaceutics17080964 (PMC12389653; doi:10.3390/pharmaceutics17080964)
Supplement: Supplementary file 1 [file pharmaceutics-17-00964-s001.zip › pharmaceutics-3735282-supplementary.pdf]

Supplementary to

Limited Efficacy of Nanoparticle-Assisted Electroporation for Membrane Permeabilization and Gene Electrotransfer

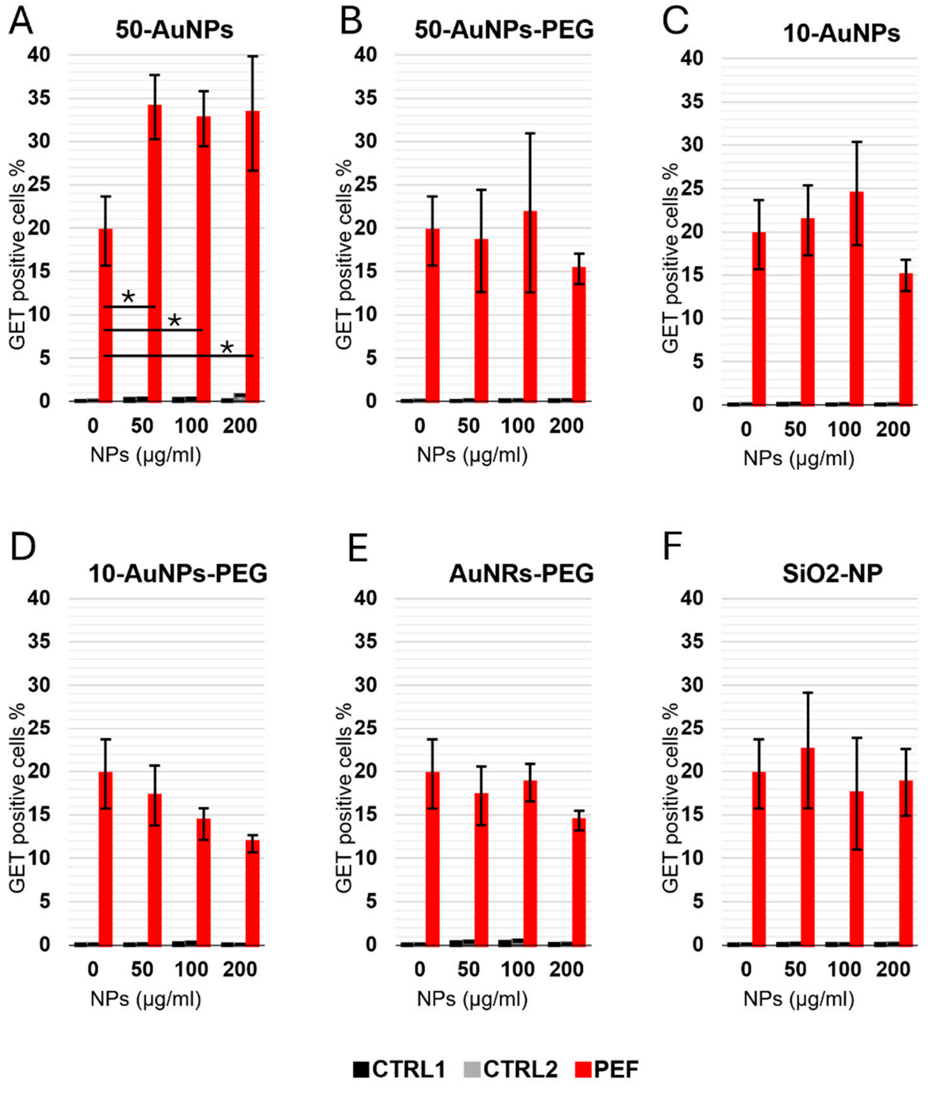

Figure S1. GFP positive cells in GET treatment on CHO cells performed with different nanoparticle concentrations. A) 50-AuNPs, B) 50-AuNPs-PEG, C) 10-AuNPs, D) 10-AuNPs-PEG E) AuNRs-PEG, F) SiO2-NP. Asterisk (*) marks a statistically significant increase. Each treatment had two controls – one with (black) and one without (gray) plasmid both in the absence of the treatment (0 V), while PEF treatment is shown with red.

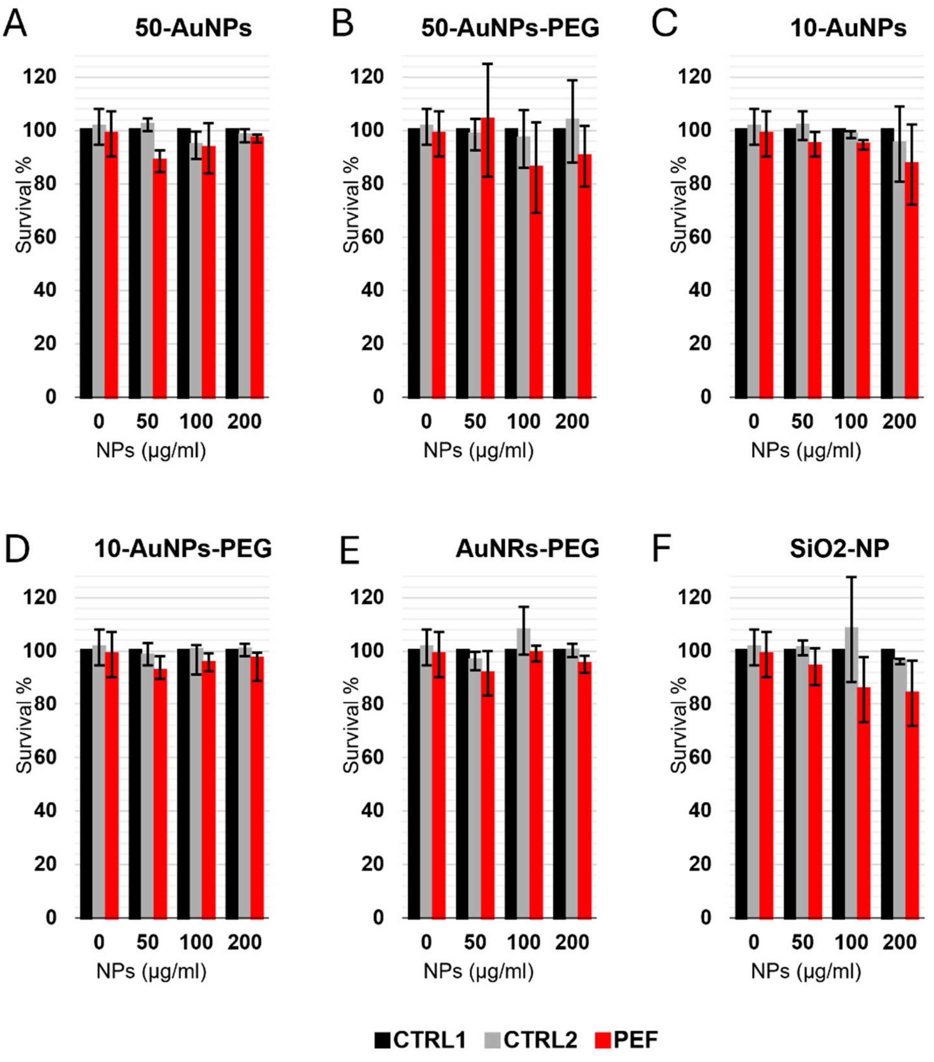

Figure S2. Survival in GET treatment on CHO cells performed with different nanoparticle concentrations. A) 50-AuNPs, B) 50-AuNPs-PEG, C) 10-AuNPs, D) 10-AuNPs-PEG E) AuNRs-PEG, F) SiO2-NP. Each treatment had two controls – one with (black) and one without (gray) plasmid both in the absence of the treatment (0 V), while PEF treatment is shown with red.
